# Modelling trends of CD4 counts for patients on antiretroviral therapy (ART): a comprehensive health care clinic in Nairobi, Kenya

**DOI:** 10.1186/s12879-021-06977-w

**Published:** 2022-01-04

**Authors:** Caroline W. Mugo, Ziv Shkedy, Samuel Mwalili, Tadesse Awoke, Roel Braekers, Dolphine Wandede, Christina Mwachari

**Affiliations:** 1 Department of Statistics and Actuarial Science, Jomo Kenyatta University of Agriculture and Technology, P.O BOX 62000, 00200 Nairobi, Kenya; 2 CENSTAT, Universitiet Hasselt, Agoralaan, 3590 Diepenbeek, Belgium; 3 Kenya Medical Research Institute, P.O BOX 54840, 00200 Nairobi, Kenya; 4 University of Gondar, Maraki 196, Gondar, Ethiopia

**Keywords:** Highly active antiretroviral therapy(HAART), HIV/AIDS, CD4

## Abstract

**Background:**

In resource-limited settings, changes in CD4 counts constitute an important component in patient monitoring and evaluation of treatment response as these patients do not have access to routine viral load testing. In this study, we quantified trends on CD4 counts in patients on highly active antiretroviral therapy (HAART) in a comprehensive health care clinic in Kenya between 2011 and 2017. We evaluated the rate of change in CD4 cell count in response to antiretroviral treatment. We further assessed factors that influenced time to treatment change focusing on baseline characteristics of the patients and different initial drug regimens used. This was a retrospective study involving 432 naïve HIV patients that had at least two CD4 count measurements for the period. The relationship between CD4 cell count and time was modeled using a semi parametric mixed effects model while the Cox proportional hazards model was used to assess factors associated with the first regimen change.

**Results:**

Majority of the patients were females and the average CD4 count at start of treatment was 362.1 $cell/mm^3$. The CD4 count measurements increased nonlinearly over time and these trends were similar regardless of the treatment regimen administered to the patients. The change of logarithm CD4 cell count rises fast for in the first 450 days of antiretroviral initiation. The average time to first regimen change was 2142 days. Tenoforvir (TDF) based regimens had a lower drug substitution(aHR 0.2682, 95% CI:0.08263- 0.8706) compared to Zidovudine(AZT).

**Conclusion:**

The backbone used was found to be associated with regimen changes among the patients with fewer switches being observed, with the use of TDF when compared to AZT. There was however no significant difference between TDF and AZT in terms of the rate of change in logarithm CD4 count over time.

**Supplementary Information:**

The online version contains supplementary material available at 10.1186/s12879-021-06977-w.

## Background

Human immunodeficiency virus (HIV) epidemic has become one of the greatest threats to human health and development. The number of persons living with HIV has risen to about 36 million with a high percentage of about $67\%$ being in Sub-Saharan Africa [1]. According to [1], Kenya has one of the largest HIV epidemic in the world with a prevalence rate of 5.9 %.

Combating the epidemic requires strategies that reduce the new infections and improvement of the survival rates of those already infected. In recent years, highly active antiretroviral therapy (HAART) has become available to the patients with World Health organization (WHO) guidelines recommending initiation of ART in all adults and adolescents with HIV [2]. The benefits of combined antiretroviral therapy are well documented in literature [3, 4]. After initiation of antiretroviral therapy(ART) most patients experience a reduction in HIV viral load combined with an increase in CD4 cell count which reduces the risk of HIV related events and death. A strong predictor of the progression to Acquired Immunodeficiency Syndrome (AIDS) is the CD4+ T-cell (CD4) count typically reported as an absolute level or count of cells (expressed as cells per cubic millimeter of blood) [5]. Changes in CD4 count constitute an important component in patient monitoring and evaluation of treatment response as these patients do not have access to routine viral load testing. WHO recommends CD4 count monitoring every 6 months and viral load testing only when the capacity exists [6]. However, the measurements of CD4 in most developing countries is not on a regular basis.

Revision of WHO guidelines in 2010 brought changes in the management of HIV infected patients among them use of less toxic antiretroviral drugs in first line [2]. In particular the recommendation emphasized moving away from the use of Stavudine (d4T) [7]. In line with these recommendations, antiretroviral therapy guidelines for Kenya in 2011 [8] suggested that first-line regimens for HIV naïve adults and adolescents consist of two nucleoside reverse trancriptase inhibitors (NRTIs) as ”backbone” along with one non-nucleoside reverse-transcrptase inhibitor (NNRTI). In the guideline, Lamivudine (3TC) was combined with one of two NRTIs and one of two NNRTI. The NRTIs were Zidovidine (AZT) and tenofovir (TDF) while the NNRTIs included efavirenz (EFV) and nevirapine (NVP). Furthermore, the treatment was to be availed to all patients unlike the previous guide where the treatment was given to patients with a CD4 count$\le 350 cell/mm^3$.

The success of HAART nonetheless critically depends on regular patient follow-up to the treatment during their lifetime. It is important to maintain the patients on first-line treatment regimens as much as possible since a second-line treatments are expensive [9]. ART drug regimens are however changed due to various reasons which include but not limited to toxicity, co-morbidity, pregnancy and treatment failure [10, 11]. These drug regimen modifications limit treatment options and introduce challenges such as monitoring and adherence difficulties among the patients. These modifications have also been associated with poor clinical outcomes [12]. According to [13], fewer drug substitutions may be important for success and control cost of managing HIV patients from a clinical stand point. It is therefore of utmost importance to understand when ART regimens are switched in order to identify drugs that can be administered in real world settings and assess factors that are associated with these treatment modifications.

In this study we aim to assess CD4 cell count trends over time for patients on combined ART in one of the comprehensive health care clinics in Nairobi, Kenya and evaluate the rate of change in CD4 count in response to antiretroviral treatment as an indicator of how fast the patients responded to treatment. In addition, the study also evaluates whether there was a difference in evolution with respect to the different NRTI and NNRTI used by the patient. Further, we estimate time to first drug regimen change and establish if it is associated with the baseline characteristics of the patients. Minimal treatment switches would be an indicator of less adverse treatment effects. In "Methods" section we discuss the methods used, the results are then displayed in "Results" section and finally a comprehensive discussion and conclusion is presented in "Discussion" section.

## Methods

### Study participants

The data used in this study was from a retrospective study sourced from the Kenya Medical Research Institute (KEMRI). In the original study Ethical review committee(ERC) permission was obtained locally and internationally; the protocol was reviewed for human subject concerns and approved by the Kenya Medical Research Institute ERC and University of California San Francisco Committee on Human Research. The study involved HIV naïve patients attending one of the comprehensive health care clinics in Nairobi, Kenya for the period between September 2011 and 2017. In this study, we included only 432 patients $\ge$18 years of age who had at least two CD4 count measurements during the follow up period and whose drug regimen was recorded. Informed consent was obtained from all participants included in the study. Baseline characteristics of the patients at initiation of ART such as patient gender, age and WHO clinical stage are also included. All methods were performed in accordance with the relevant guidelines and regulations.

### Treatments

According to [8], the forth edition of the ART Kenya guidelines released in December 2011 the recommended first-line regimens for naïve adults and adolescents consisted of two nucleoside reverse transcriptase inhibitors (NRTIs) as ”backbone” along with one non-nucleoside reverse-transcriptase inhibitor (NNRTI). In the giudeline, Lamivudine (3TC) was combined with one of two NRTIs and one of two NNRTI options implying that we have four treatment combinations for first line HAART. The NRTIs were zidovudine(AZT) and tenofovir (TDF) while the NNRTIs included efavirenz (EFV) and nevirapine (NVP).

As stated earlier in the previous guideline [14] Stavudine(d4T) was used as an NNRTI. In this study we excluded any patients who were on d4T as it was being phased out. During the follow up period some of the patients changed regimens for different reasons. These changes in regimen involved either the backbone or NNRTI. Whilst the number of changes made by a patient may be more than one,in this study we considered the first regimen change as our outcome of interest. The time to first treatment change was calculated by subtracting the date of ART initiation from the date of ART modification.

### Statistical analysis

Exploratory data analysis and descriptive statistics was carried out to give an insight into the data. Baseline categorical variables were cross tabulated to give the proportions in different categories and a Chi-square test performed to find out if any association existed among the variables.

### Time to treatment change

For the purpose of this study treatment change/modification was defined as the changing of at least one ART agent in the regimen within first line therapy. Patients were censored if treatment change was not observed until their last visit to the clinic. This was done for patients who were lost to follow up and for those still alive at the end of study. Time to treatment change was estimated using the Kaplan-Meier estimator. The log-rank(LR) test was used to compare between groups of baseline characteristics and initial treatment administered to the patients. The baseline characteristics that were considered were the sex of the patient (male versus female), NRTI administered (3TC Plus AZT versus 3TC plus TDF) and the NNRTI agent (NVP versus EFV).

The Cox regression model [15] was used to identify the baseline characteristics that could be associated with first treatment or regimen modification. The hazard ratio with 95% confidence interval was used to test statistical significant association between time to first treatment change and the patient’s baseline characteristics. Details of the Cox regression model are in Additional file 1.

### Analysis of CD4 cell count

CD4 cell counts were log-transformed to meet the assumption about stability of the variance with increasing CD4 cell count. Individual trajectory plots were obtained to give an indication of how the patient’s CD4 count evolved over time. We constructed a mean profile of the log transformed CD4 over time in months. Further, the average profile plots were fitted for different baseline characteristics.

A simple parametric model may be adequate to describe subject-specific profiles in terms of random effects [16]. However, the relevance of normality assumption on random effects may be questionable. Furthermore, the individual profiles are non-linear making parametric models too restrictive [17]. We propose a data-driven approach based on semi-parametric regression models [18, 19]. In this approach, a patient specific random intercept is used to capture the correlation of CD4 cell count measurements over time within the patients. We assume patient specific random parameters for both linear and quadratic time effects to capture different evolution patterns between the patients. This model allows smoothing with respect to time. The link between mixed model and smoothing provides a flexible framework for estimating profiles in a data driven way [20]. First order derivatives for each treatment groups were obtained and plotted with 95% confidence band to determine the effect of treatments on the rate of change in the logarithm of CD4 cell count over time. The rate of change is helpful in assessing the effectiveness of treatment administered to the patients. A more detailed model formulation is attached in the supporting materials(Additional file 1).The proposed model was fitted using the *gamm* procedure available in R package *mgcv* [21].

## Results

### Baseline characteristics

A total of 432 patients with at least two measurements of CD4 count were included in the study. Table 1 provides a summary of the CD4 count measurements and age of the patients. Subjects were followed up for a maximum of 2149 days. The median time of follow up was green 405 days. The number of CD4 count measurements per subject ranged from two to fifteen with a median of 6 measurements. A majority of the patients (55.6%) had CD4 cell count of less than 350 cells/mm^3^ which was the previous cutoff point to start ART. Even with majority having low CD4 count the average CD4 count at the start of ART was 362.1 cells/mm^3^. These average CD4 cont values fluctuated over time as shown in Table 5. The highest CD4 count being 1631 with a median value of 397.

**Table 1:** Summary continuous characteristics

Variable	Minimum	Q1	Median	Mean	Q4	Maximum
Age	18	36	42	42.6	49	69
CD4	1	283	405	413.5	533.2	1631.4
Log CD4	0	5.64	6.00	5.543	6.27	7.397
Observation time in days	0	85	244	373.7	548.2	2149
No of CD4 measurements	2	4	6	5.7	7	15

Categorical baseline characteristics are summarized in Table 2. Most patients in the study were females at 66.9%. At initiation of ART 65.0% of the patients were at WHO clinical stage II.

**Table 2:** Summary of categorical baseline characteristics

Variable	Categories	Count	Percentage (%)
Gender	Female	289	66.9
	Male	143	33.1
WHO stage	Stage 1	54	12.5
	Stage 2	281	65.0
	Stage 3	58	13.4
	Stage 4	9	2.1
	Unknown	30	7.2

With reference to drug regimens, a majority of the patients were on Lamivudine + Tenofovir + Efavirenz (58.8%). Efavirenz was the most utilized among the NNRTI at 65.3% while Tenofovir was the most used NNRT at 64.6% as shown in Table 3.

**Table 3:** Treatments at baseline

Backbone	EFV(%)	NVP(%)
3TC TDF	254 (58.8)	25 (5.8)
3TC AZT	28 (6.5)	125 (28.9)

**Fig. 1:**
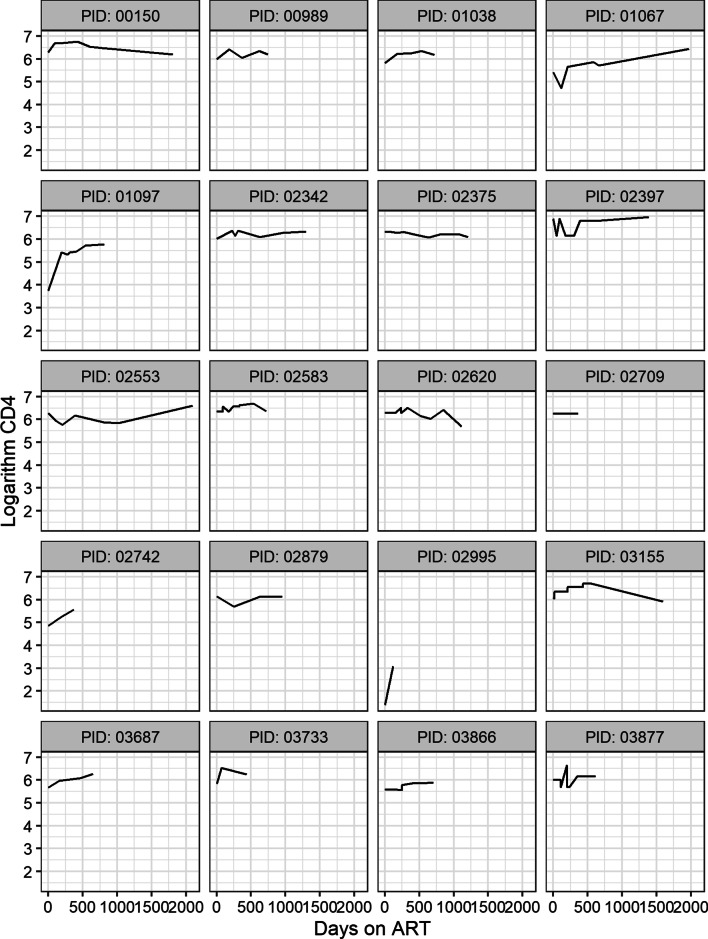
Individual Profiles of logarithm CD4 cell count over time

A sample of twenty individual profiles of the patients are presented in Fig. 1. From the profiles we observe that there is within and between subject variability. The subjects start at different baseline CD4 counts and evolve differently over time. There is an indication that the overall trend is not linear over time. Initially most patient’s logarithm CD4 count increases rapidly then stabilizes.

### Time to treatment change

**Fig. 2:**
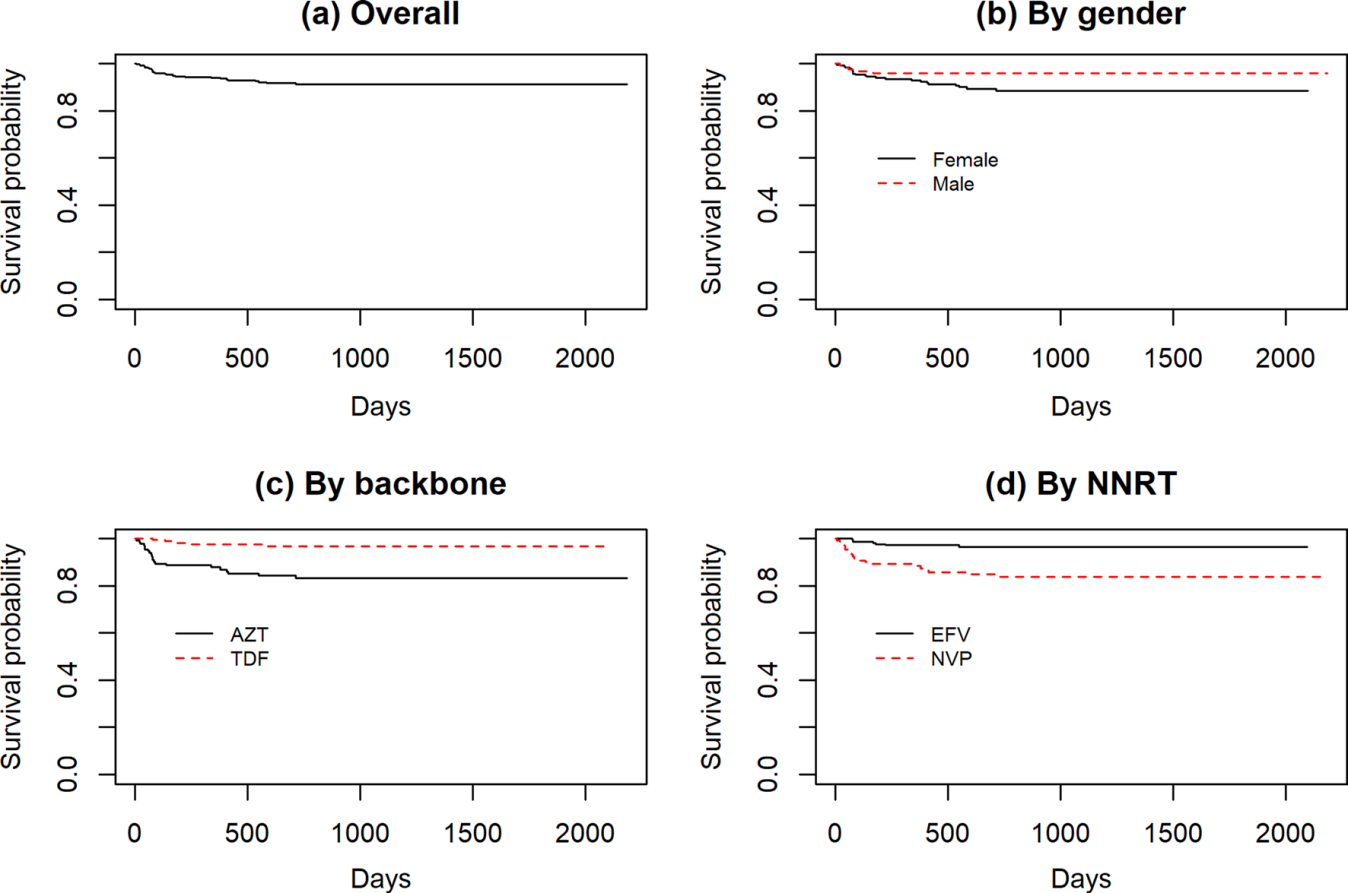
Kaplan–Meir curves for time to treatment change by different baseline characteristics. **a** Overall, **b** by gender, **c** by NRTI, **d** by NNRT

The number of patients that had at least one treatment change account for about 7.5% of the patients. The Log-rank test was used to test the difference between categories of baseline covariates with the probability of treatment modification. This test revealed the presence of significant difference among the categories of baseline NNRTI, NRTI and gender. There was no significant difference for WHO clinical stage. The Kaplan–Meir curves are shown in Fig. 2. The survival curve for time to treatment change shows steady increase on overall. The estimated median time to first treatment change was not reached since there were few regimen changes. The average time to treatment change was estimated to be 2141 days.

The cox regression analysis results are presented in Table 4. Adjusting for the baseline characteristics we find that only the backbone was associated with the drug regimen changes (aHR = 0.2682 (95% C.I: 0.08263$-$ 0.8706e)) which was also observed in the log-rank test. Patients initiated on Zidovudine were at a higher risk of changing treatment compared to those on Tenofovir. The unadjusted analysis show that males were less likely to have treatment modification compared to females. This result was however only observed in the unadjusted model and was not in the adjusted analysis.

**Table 4:** Cox-regression analysis of factors associated with time to treatment change

Variable	Categories	UHR(95$\%$CI)	AHR(95 $\%$CI)	p-value
Gender	Female	1	1	
	Male	0.3977 (0.1512$-$ 1.046e)	0.5524 (0.20444 $-$ 1.4925e)	0.2419
NNRTI	Efavirenz	1	1	
	Nevirapine	4.926 (2.082$-$ 11.66e)	1.8107 (0.58091$-$ 5.6438e)	0.3060
Backbone	Zidovudine	1	1	
	Tenofovir	0.1693 (0.06829$-$ 0.4198e)	0.2682 (0.08263$-$ 0.8706e)	0.0285

**Table 5:** Average CD4 count over time in months

	Month	Average CD4
1	0.00	362.12
2	6.00	410.13
3	12.00	439.47
4	18.00	465.39
5	24.00	480.10
6	30.00	421.42
7	36.00	422.18
8	42.00	605.25
9	48.00	602.67
10	54.00	571.29
11	60.00	550.67
12	66.00	587.50
13	72.00	905.00

### Modelling CD4 cell count

**Fig. 3:**
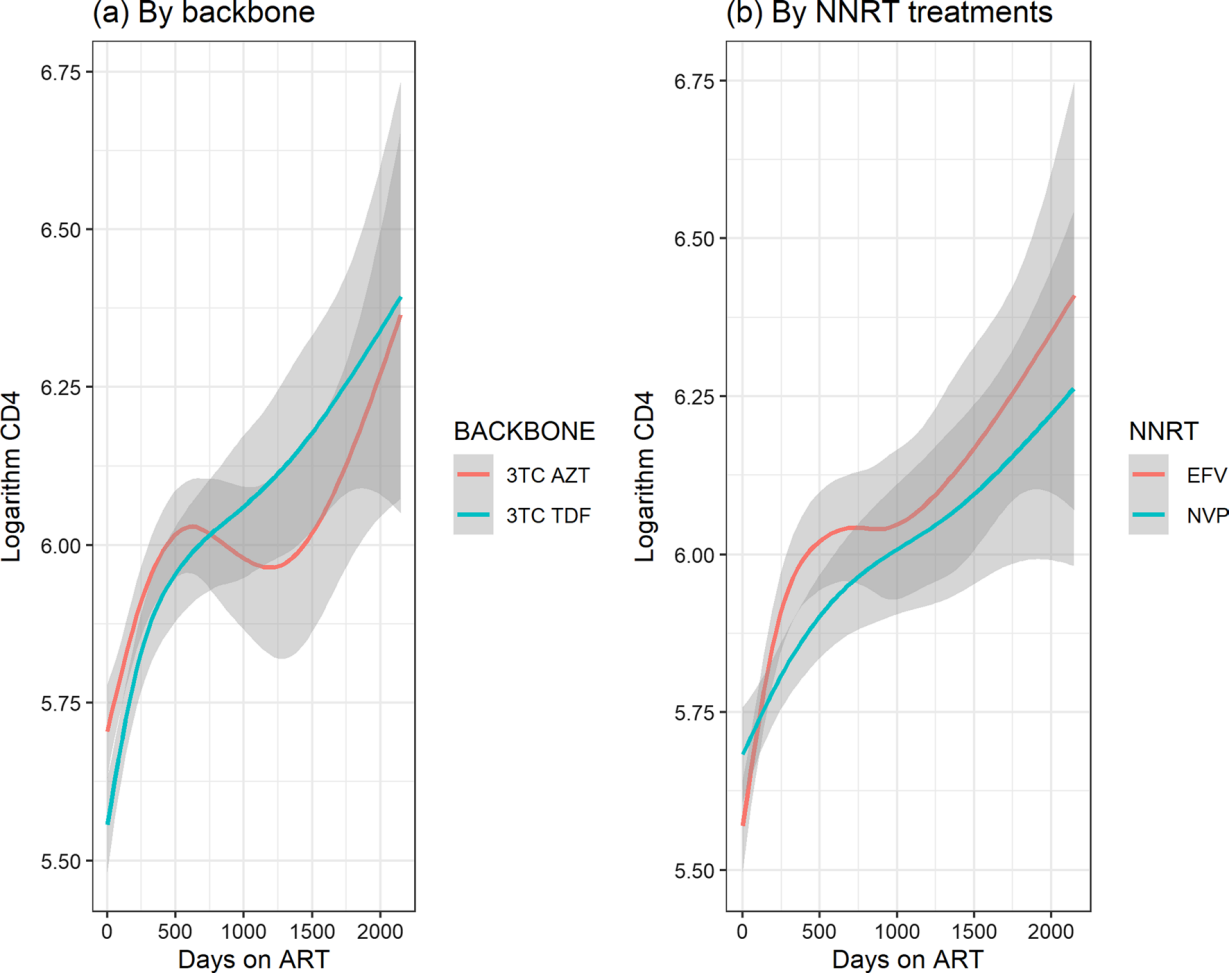
Graph of logarithm CD4 count over time in days. **a** By backbone, **b** By NNRT treatments

**Fig. 4:**
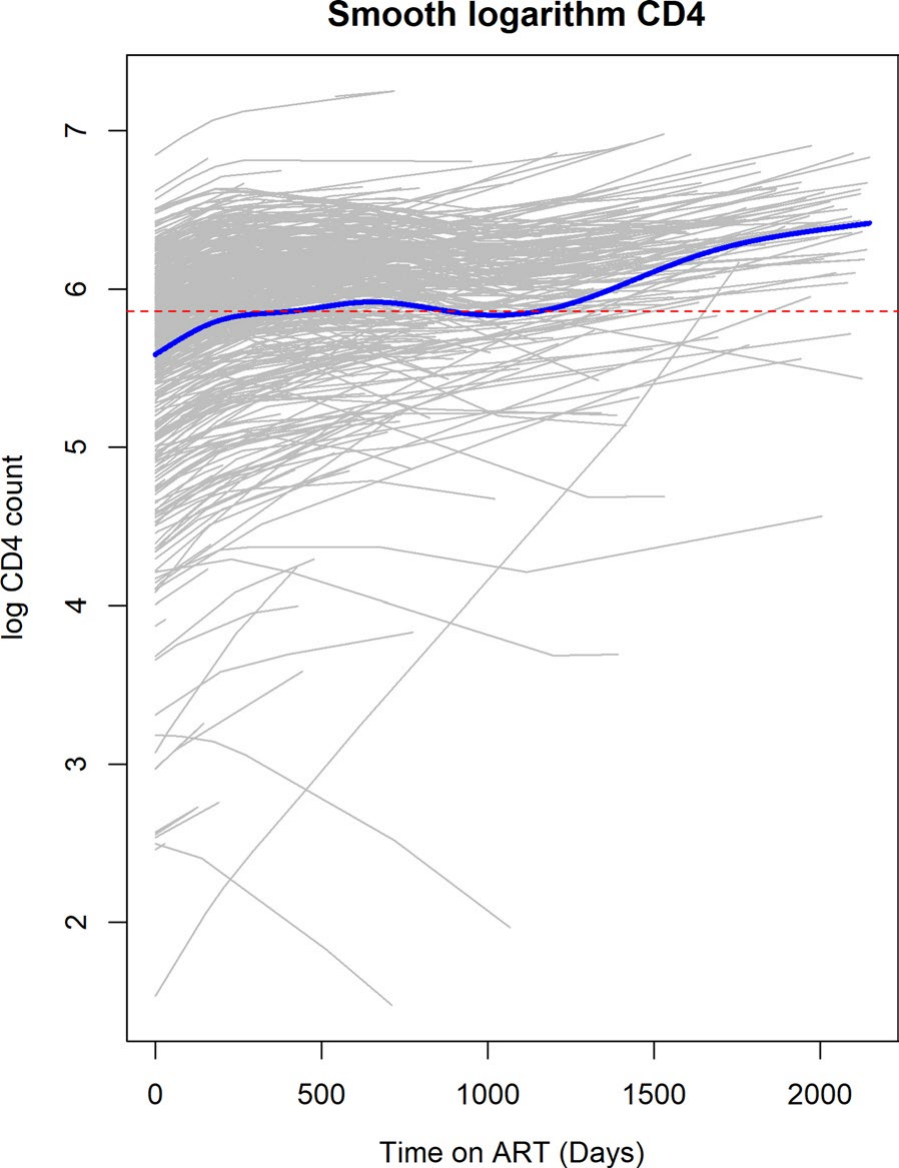
Fitted individual CD4 count profiles with average smoothed line over time

The logarithm CD4 count trend is the same for both NNRTI treatments as seen in Fig. 3. After initiation to ART the rise of logarithm CD4 count in patients on Efavirenz is faster compared to those on Nevirapine. At later time points we observe that the logarthim CD4 cell count of those taking EFavirenz is higher than for those on Nevirapine. However, there appears to be no difference between the trend for TDF and AZT. The fitted individual profiles for the patients and overall average trend of logarithm CD4 count from semiparametric model is shown in Fig. 4. There was a rise in the logarithm CD4 cell count in the first days after initiation of ART and thereafter it stabilizes.

**Fig. 5:**
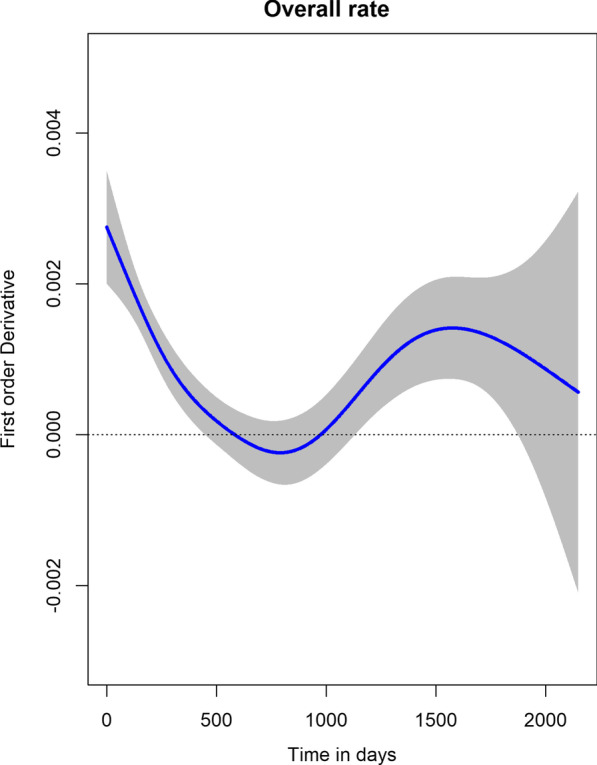
Estimated rate of change of logarithm CD4 cell count over time

The first order derivative of the semiparametric mixed model fitted allows estimation of the rate of change in CD4 counts. A derivative equal to zero implies a constant trend with respect to time. The Fig. 5 presents the rate of logarithm CD4 change over time with the 95% confidence band. It was observed that the rate decreases to zero in the first 450 days after initiation to ART on average and thereafter remains close to zero. However, a closer observation on the individual profiles indicated that not all subjects got to zero. The confidence band was wide towards the end of study.

**Fig. 6:**
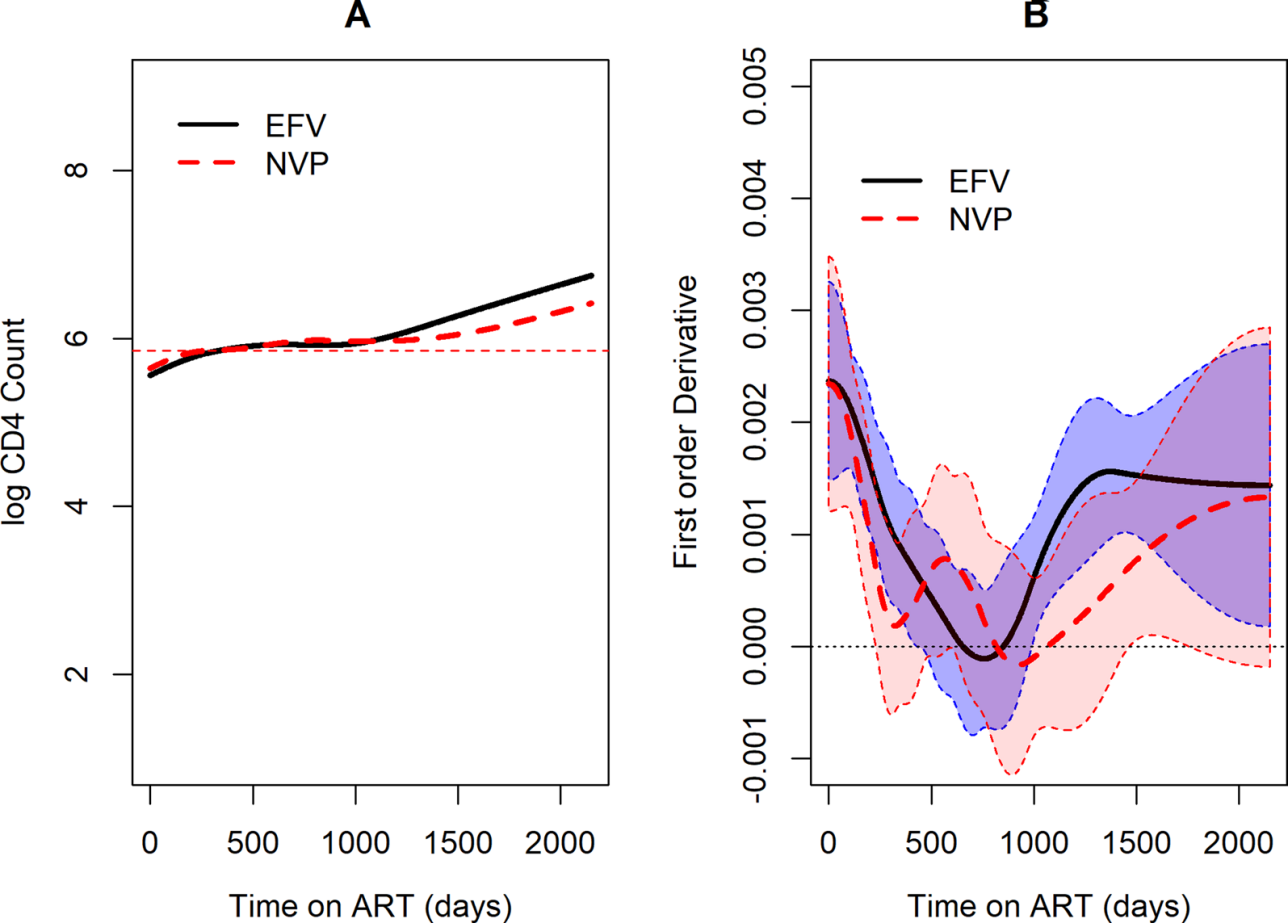
**A** Predicted logarithm CD4 and **B** Estimated rate of change of log CD4 over time by NNRTI

**Fig. 7:**
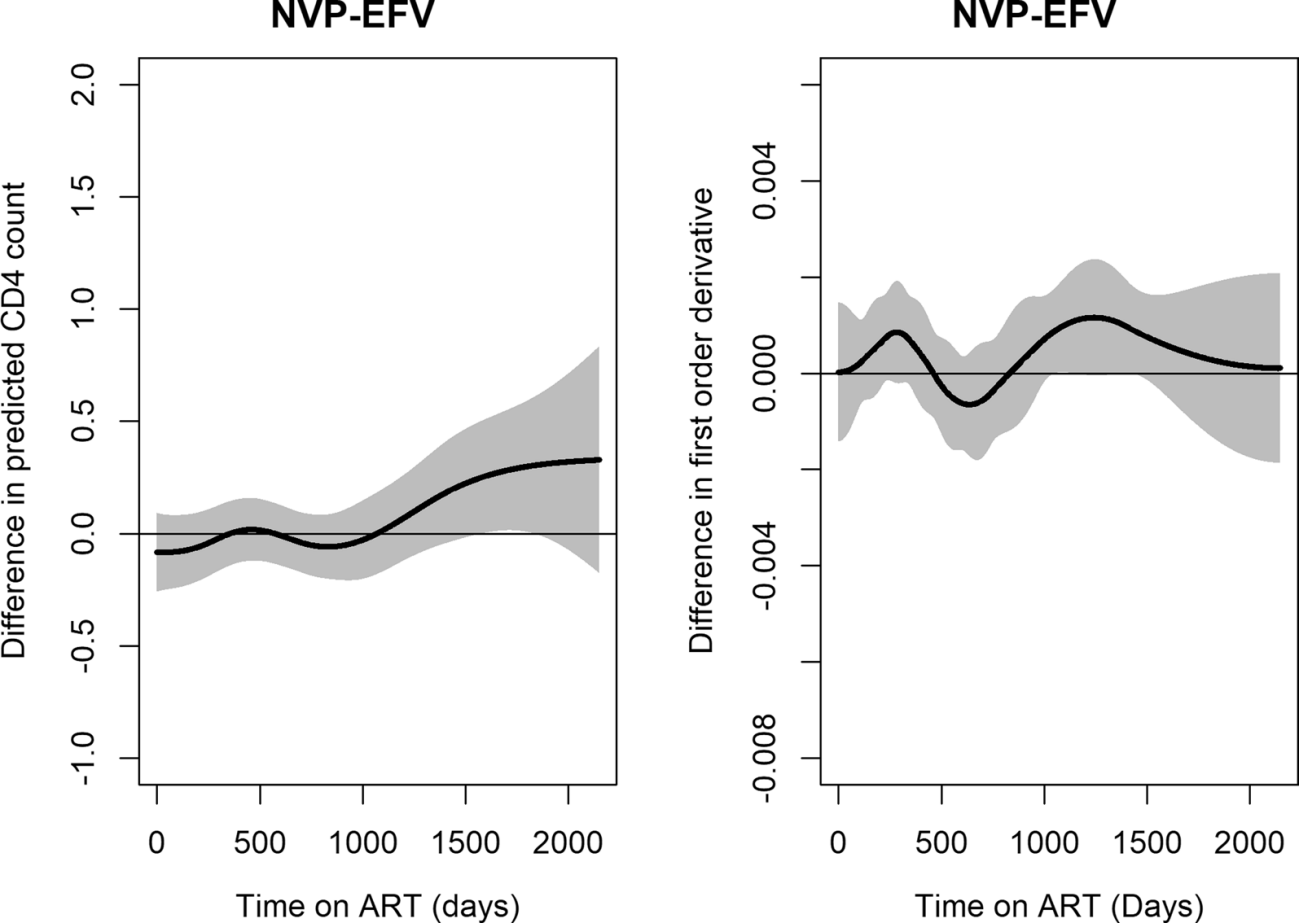
Estimated difference in predicted log CD4 cell count and rate of change in CD4 cell count over time between EFV and NVP

**Fig. 8:**
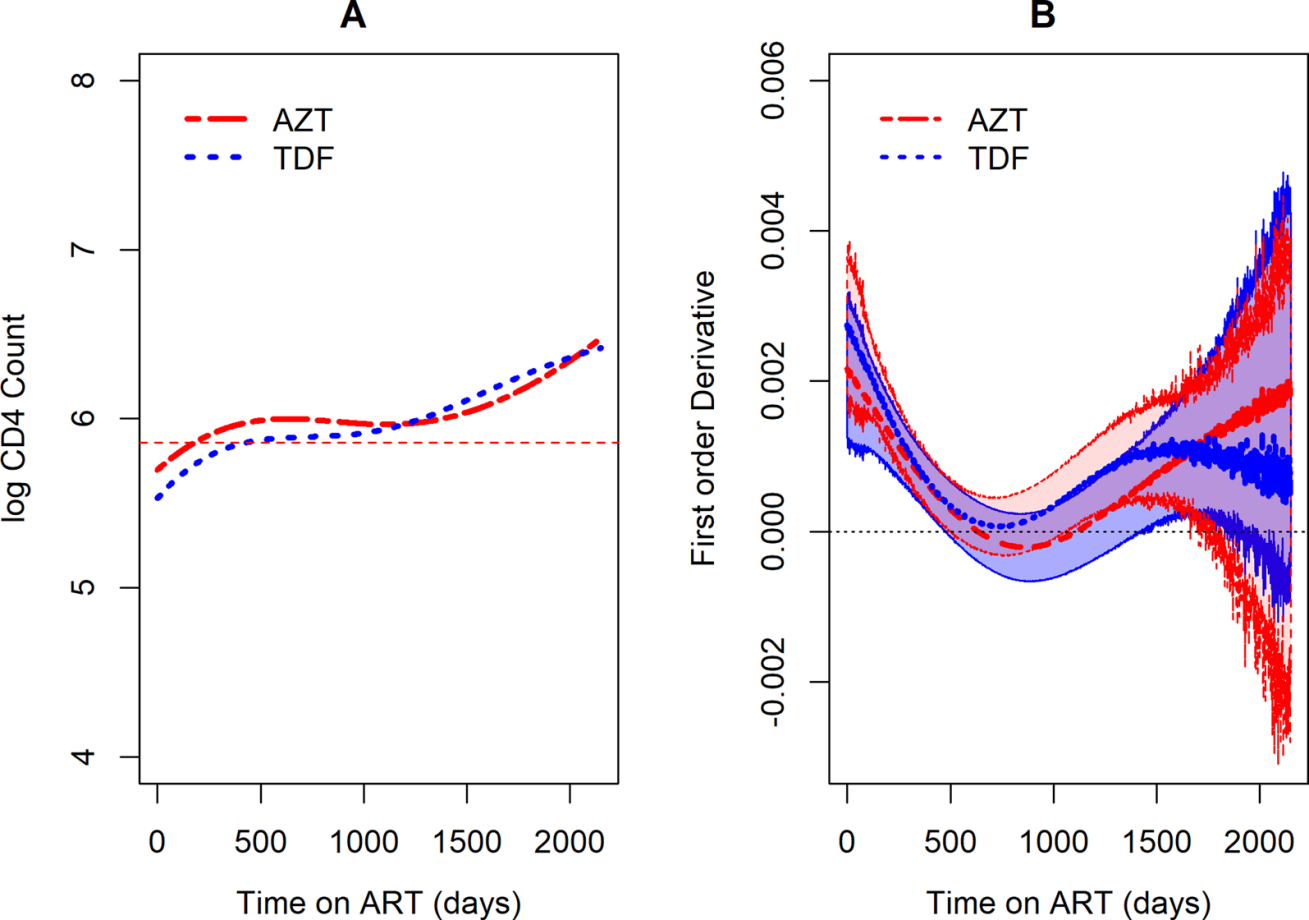
**A**Predicted logarithm CD4 and **B** Estimated rate of change of logarithm CD4 cell count over time by backbone

**Fig. 9:**
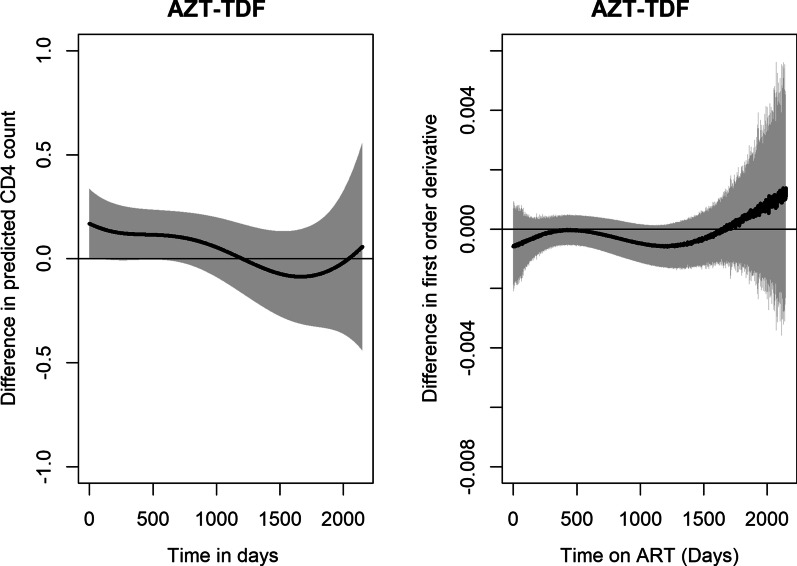
Estimated difference between the predicted log CD4 cell count over time by NRTI backbone

The model allows comparison between different groups. The treatment response for the two NNRTI drugs was the same for both EFV and NVP in the evolution of logarithm CD4 cell counts as shown in Fig. 6A and the rate of change Fig. 6B in the left panel and right panel respectively. Figure 7 displays the difference in estimated rate of change between NVP and EFV. The 95% confidence band covers zero for most part of the study an indication that there was no difference in the evolution of CD4 cell counts for patients taking either of the treatments. However at the end of the study, we observe that the value is greater than zero with a wide confidence band.

Similarly, the trend of logarithm CD4 cell counts was estimated for the backbones TDF and AZT. The trends rise steadily for both NRTIs over time with differences observed at initiation of ART. AZT had higher logarithm CD4 cell counts over time compared to TDF as seen in Fig. 8A. A wider confidence band was observed towards the end of study with regard to the rate of change Fig. 8B. A further investigation on the difference between estimated curves by the backbones AZT and TDF, showed that even though there were differences at initiation of treatment, thereafter there were no observable differences as shown in Fig. 9.

## Discussion

Analysis of the CD4 count is an important component in monitoring and evaluating progression of HIV in resource limited settings. This study aimed at describing the evolution of CD4 cell counts and evaluating time to first treatment change among HIV patients after initiation of ART. Majority of patients in the study were in WHO clinical stage II. The number of female patients was higher compared to males which could be explained by the fact that some patients were referrals from the Antenatal care (ANC) clinics. The evolution of CD4 count increases nonlinearly over time with rapid increase in CD4 cell count observed immediately after initiation of ART which then stabilizes with time. The change in CD4 count rises fast in the first 450 days of ART initiation which is slightly more than a year. This was longer than in the study conducted in Ethiopia [19] where the rapid increase was observed in the first 10 months. The trend of logarithm CD4 cell count over time was the same regardless of the NNRTI treatment administered to the patients.

In line with previous studies, majority of the females were observed to have changed their treatment compared to males [10, 11, 22]. From this study though males were less likely to have their drug regimens changed (adjusted hazard ratio 0.5524, 95% CI: 0.2044$-$ 1.4925e) compared to the females, this was not significant. This agrees with a study by [23] which showed no significant differences in treatment modification time by gender and regimen. Most of the changes were mainly from EFV to NVP which could be explained by the fact that EFV is not recommended for use by pregnant women [8, 14]. However, a study by [24] indicated that the use of NVP-based regimens seemed to be associated with higher risk of modifications compared to use of EFV.

Comparing the backbone, Tenofovir (TDF)based regimens had a lower drug substitution (aHR 0.2682, 95% CI: 0.0826$-$ 0.8706e) compared to zidovudine(AZT). This result concurs with a study conducted by [25] in Ethiopia which concluded that TDF based regimens have more efficacy than AZT based regimens. Another study in Kenya [26] showed that TDF had lower modifications of the ART treatments. Further, a study in South Africa [13] showed that TDF seemed to perform better notably with less drug substitution. On the contrary, a study by [27] in the largest Kenyan referral indicated that AZT based regimens had a better performance across all aspects of health related quality of life. The trend in CD4 recovery for TDF and AZT is similar with little difference observed at the initiation of ART.

A comparison of Efavirenz and Nevirapine in the evolution of CD4 count indicated no differences. This is consistent with findings of in the study by [28] who found no significant differences among the study groups in the proportions with increases in CD4-positive cells. A systematic review by [29] also shows that EFV and NVP have similar benefits in initial treatment of HIV infection when combined with two NRTIs. Other studies conducted to compare the treatments have however indicated that EFV was better than NVP. For instance, studies by [30] showed that patients on EFV recovered more CD4 cell counts than those on NVP while [31, 32] concluded that EFV-containing antiretroviral regimens were associated with superior clinical outcome, as measured by time to treatment failure. Another study by [33] indicated that EFV based ART regimen was less likely to lead to virological failure compared to NVP.

This study however had several limitations. First, the results may not be generalizable to all the clinics in Kenya since switching strategies may be different among hospitals. Secondly, patients attending the comprehensive health care clinic were mainly referrals from antenatal care clinics and voluntary counseling centers and therefore may not completely represent all the HIV patients in Kenya. Thirdly, the study was retrospective and therefore data was recorded for other purposes, data unavailability, including reasons for regimen change or adherence to ART regimens limits further interpretation of results. CD4 cell count measurement was not performed at particular time points and the number of measurements differ from the different patients. In addition, the analysis of drug regimen substitution was restricted to first treatment change only yet there were patients who changed treatment more than once. Taking into account all the drug changes may reveal a different finding.

In conclusion, the study revealed that the evolution of CD4 count does not depend on the NNRTI administered to the patient since the trend of EFV and NVP containing elements are comparable. There were observed differences in the NRTI backbones especially at the initiation of ART which was not significant. In addition, only the backbone NRTI was associated with regimen changes with fewer switching being observed for patients on TDF.

## Supplementary Information

**Additional file 1.** Supplementary appendix.

## Data Availability

The datasets during and/or analysed during the current study available from the corresponding author on reasonable request to be considered by the lead investigator.
