# Supplementary material for: Modelling trends of CD4 counts for patients on antiretroviral therapy (ART): a comprehensive health care clinic in Nairobi, Kenya
Source: BMC Infect Dis. 2022 Jan 4;22:29. doi: 10.1186/s12879-021-06977-w (PMC8725499; doi:10.1186/s12879-021-06977-w)
Supplement: Supplementary file 1 — Additional file 1. Supplementary appendix. [file 12879_2021_6977_MOESM1_ESM.pdf]

# Supplementary appendix to " Modelling trends of CD4 counts for patients on antiretroviral therapy(ART):A comprehensive health clinic in Nairobi, Kenya." Caroline Mugo et al.

## 1 Time to event statistical analysis

To model the time to first treatment change the Cox regression model was used [1].The model was formulated as;

$$h_i(t|x_i) = h_0(t)exp(X_i\beta) \tag{1}$$

where $h_0(t)$ is a baseline hazard function that describes the risk for individuals with $X_i = 0$ and $exp(X_i\beta)$ is the relative risk representing a proportionate increase or reduction in risk, associated with a set of characteristics $X_i$. Suppose we let $T$ be a random variable representing failure time in our case time to regimen or treatment switch. The probability of failure time occurring at exactly time $t$ can be formulated as:

$$f(t) = lim_{h\to 0}\frac{P[t \leq T < t+h]}{h} \tag{2}$$

The cumulative distribution of the random variable $T$ is as shown

$$F(t) = P(T \leq t), t > 0,$$

$$S(t) = P[T \geq t] = 1 - F(t^{-})$$

The hazard function

$$\lambda(t) = lim_{h\to 0}\frac{P[t \leq T < t+h|T \geq t]}{h} \tag{3}$$

The log-rank(LR) test[1] was used to compare between groups of baseline characteristics and initial treatment administered to the patients.The statistic is given by

$$LR = \frac{\sum_{i=1}^{k}(E_i - O_i)^2}{E_i} \tag{4}$$

where $O_i$ is the observed number of failures and $E_i$ is the expected number of failures. Under the null hypothesis that the survival distribution are the same for the groups, then the log-rank test statistic has a chi-square distribution.

## 2 Statistical Analysis of CD4 count

Let $y_{ij}$ denote the response for subject $i$ measured at occasion $j$. Further if we let $\mathbf{y}_i$ denote a vector of all repeated measurements for subject $i$ then we can formulate the general linear mixed effects model as:

$$\mathbf{y}_i = X_i\beta_i + Z_i\mathbf{b}_i + \epsilon_i \tag{5}$$

where $\mathbf{y}_i$ ,i=1,2,3...,$n_i$ is a $n_i$-dimensional vector of the log transformed CD4 counts for patient $i$ at time $j$,$X_i$ and $Z_i$ are $n_i \times p$ and $n_i \times q$ matrices of known covariates,$\beta$ is a p-dimensional vector of fixed effects and **b** q-dimensional vector of subject specific random effects and $\epsilon_i$ is the residual component .
Many biomedical experiments generate non-linear data and imposing a parametric function for the mean evolution over time may yield unsatisfactory results[2].The individual CD4 count profiles for HIV patients are non-linear implying that the parametric models may be restrictive.Moreover, the repeated measurements will result in correlated values within a subject.The model we propose is a data driven approach based on semi-parametric regression models[3][4]. In the proposed model, patient-specific random intercept is used to capture correlation of CD4 count over time within the patient. We also assume patient-specific random parameters for both the linear and quadratic time effects to capture the different evolution patterns for the patients' CD4 count over time. The semi-parametric mixed effects model with patient specific random effects can be expressed as

$$\mathbf{Y}_i(t_i) = S(t_i) + b_{0i} + b_{1i}t_i + b_{2i}t_i^2 + \epsilon_{it_i} \tag{6}$$

Where $S(t_i)$ is the non-parametric component of the model.The patient specific random effects are assumed too follow a multivariate normal distribution, $[b_{0i}, b_{1i}, b_{2i}]^T \tilde{MVN}(0, \sum_b)$ where $\sum_b$ denotes the variance covariance matrix of patient specific random effects. The residuals are assumed to be normally distributed with mean zero and variance $\sigma_E^2$.
S(t) is a smoother to the logarithm CD4 evolution over time.This smoother can be expressed as

$$S(t_i) = \beta_0 + \sum_{l=1}^{v} \beta_l f_l(t_i) \tag{7}$$

where $f_l(t_i)$ are a set of thin plate splines basis functions[2][5] and $\beta_l$ are coefficients of the basis functions.

### Confidence intervals

Let $\beta$ be a parameter vector containing all fixed and random effects for the smooth terms, $X_i$ is the corresponding covariates , $Z_i$ is the design matrix for the random effects, and D is the covariance matrix for the random effects . The penalized thin plate spline model can be expressed as a mixed model of the form

$$\mathbf{Y}_i = \mathbf{X}_i\beta + Z_i b_i + \epsilon_{it_i} \tag{8}$$

For the given values of the parameters associated with the random effect and error application of maximum likelihood and Best prediction BLUP estimate for $S$ is given by

$$\hat{S} = X\hat{\beta} \tag{9}$$

A point-wise confidence interval for the average fitted problem can be obtained by

$$\hat{S}_t \pm t_{1-\frac{\alpha}{2}} s.d(\hat{S}(t)) \tag{10}$$

Where $s.d(\hat{S}(t)$ is the square root of the diagonal of the variance covariance matrix .

## Pairwise comparison of treatment groups

The model formulated in equation 6 allows us to incorporate variables.Therefore, we can make comparison between groups such as the different NNRTs and NRTIs. This implies that we can investigate if there is a difference in the treatment groups by comparing their average profiles over time. The model can be formulated as

$$\mathbf{Y}_{ij}(t_i) = \underbrace{\beta_{0g} + \beta_1 treat_g + \sum_{i=1}^{v} \beta_{ig} f_i(t_i)}_{s_g(t)} + b_{0i} + b_{1i}t_i + b_{2i}t_i^2 + \epsilon_{it_i} \tag{11}$$

where $Y_{ij}$ is the response for the $i^{th}$ subject in treatment $j$ at time point $t_i$, $S_g(t)$ is a treatment group specific smoother, and $f_i(t_i)'s$ are a set of thin plate basis functions , $\beta_{ig}$ are the coefficients of the basis function. From the fitted penalized thin plate regression we estimated the spline coefficient variances for each treatment group $g$ to obtain the first order derivative.Differences in the groups were tested using the first order derivative of 11 with respect to time $t$ to obtain

$$\frac{dS_g(t_i)}{dt} + b_{1i} + 2b_{2i} \times t \tag{12}$$

Using the first order derivative of the penalized thin plate regression in 12, we can construct point-wise confidence interval in a similar way was we did for the curve.

# References


[1] Cox, D.: Regression models and life tables (with discussion). Journal of the Royal Statistical Society.Series B (Methodological) **34**(2), 187–220 (1972)

[2] Wood, S.: Generalized Additive Models: an Introduction with R. Texts in Statistical Science. Chapman & Hall, UK United Kingdom (2006)

[3] Carroll, R., Ruppert, D., Stefanski, L., Crainiceanu, C.: Measurement Error in Nonlinear Models: A Modern Perspective, Second Edition. CRC Press, United States (2006). Publisher Copyright: © 2006 by Taylor & Francis Group, LLC.

[4] Awoke, T., Worku, A., Kebede, Y., Kasim, A., Birlie, B., Braekers, R., Zuma, K., Shkedy, Z.: Modeling outcomes of first-line antiretroviral therapy and rate of cd4 counts change among a cohort of hiv/aids patients in ethiopia: A retrospective cohort study. PLoS ONE **11**(12) (2016)

[5] Ramsay, J., Silverman, B.: Functional Data Analysis, 2nd edn. New York:Springer, ??? (2005)
